# Grasping Molecular Biology Mechanisms to Optimize Plant Resistance and Advance Microbiome Role Against Phytonematodes

**DOI:** 10.3390/ijms27041744

**Published:** 2026-02-11

**Authors:** Mahfouz M. M. Abd-Elgawad

**Affiliations:** Plant Pathology Department, National Research Centre, El-Behooth St., Dokki, Giza 12622, Egypt; mahfouzian2000@yahoo.com

**Keywords:** acquired resistance, biotic/abiotic factors, molecular plant defense, yield

## Abstract

Plant-parasitic nematodes (PPNs) cause big crop losses globally. Safe/reliable methods for their durable management strategies can harness various beneficial relationships among the plant immune system and related microbiomes. Molecular mechanisms basic to these relations reveal wide arrays of significant roles for plant-healthy growth. This review focuses on such relations of microbiomes to prime and immunize plants against PPNs. It also highlights molecular issues facing PPN-resistant varieties with possible solutions such as genetic breeding/engineering, grafting, PPN-antagonistic root exudates, and novel resistant cultivars. These issues call for optimal uses of various widespread groups of microbiomes. Related plant signaling hormones and transcription factors that regulate gene expression and modulate nematode-responsive genes to ease positive/negative adaptation are presented. Exploring PPN-resistance genes, their activation mechanisms, and signaling networks offers a holistic grasp of plant defense related to biotic/abiotic factors. Such factors relevant to systemic acquired resistance (SAR) via plant–microbe interactions to manage PPNs are stressed. The microbiomes can be added as inoculants and/or steering the indigenous rhizosphere ones. Consequently, SAR is mediated by the accumulation of salicylic acid and the subsequent expression of pathogenesis-related genes. To activate SAR, adequate priming and induction of plant defense against PPNs would rely on closely linked factors. They mainly include the engaged microbiome species/strains, plant genotypes, existing fauna/flora, compatibility with other involved biologicals, and methods/rates of the inoculants. To operationalize improved plant resistance and the microbiome’s usage, novel actionable insights for research and field applications are necessary. Synthesis of adequate screening techniques in plant breeding would better use multiple parameters (molecular and classical ones)-based ratings for PPN-host suitability designation. Sound statistical analyses and interpretation approaches can better identify genotypes with high-level, stable resistance to PPNs than the commonly used ones. Linking molecular mechanisms to consistent field relevance can be progressed via dissemination of many advanced techniques. The CRISPR/Cas9 system has been effective in knocking out both the *OsHPP04* gene in rice to confer resistance against *Meloidogyne graminicola* and the *GhiMLO3* gene in cotton to minimize the *Rotylenchulus reniformis* reproduction. Its genetic modifications in crops synthesized “transgene-free” PPN-resistant plants without decreased growth/yield. Characterizing microbiome species/strains needed to prime and immunize plants requires better molecular tools for fine-scale taxonomic resolution than the common ones used. The former can distinguish closely related ones that exhibit divergent phenotypes for key attributes like stability and production of enzymes and secondary metabolites. As PPN-control strategies via tritrophic interactions are more sensitive to the relevant settings than chemical nematicides, it is suggested herein to test these settings on a case-by-case basis to avoid erratic/contradictory results. Moreover, expanding the use of automated systems to expedite detection/count processes of PPN and related microbes with objectivity/accuracy is discussed. When PPNs and their related microbial distribution patterns were modeled, more aspects of their field distributions were discovered in order to optimize their integrated management. Hence, the feasibility of site-specific microbiome application in PPN–hotspot infections can be evaluated. The main technical challenges and controversies in the field are also addressed herein. Their conceptual revision based on harnessing novel techniques/tools is direly needed for future clear trends. This review also engages raising growers’ awareness to leverage such strategies for enhancing plant resistance and advancing the microbiome role. Microbiomes enjoy wide spectrum efficacy, low fitness cost, and inheritance to next generations in durable agriculture.

## 1. Introduction

The global population increase warrants a substantial boost in crop productivity for food security. Due to increased losses in crop production caused by plant-parasitic nematodes (PPNs), their management is progressively demanding further optimization [1]. Admittedly, PPNs are the most spreading and harming soil-borne pests [2]. They mostly cause out-of-sight subterranean infections and non-specific symptoms. Therefore, the related interspecific interactions in the plant rhizosphere and crop losses are frequently unknown or largely underestimated. Obviously, regulatory constraints on nematicides and the growing importance of host resistance and microbiome-based strategies are unmistakable benchmarks to accelerate perfecting crop production without further nematicidal pollution [3]. They necessitate developing PPN plant resistance and fortifying plant immune systems that rank high among benign and reliable alternatives to long-used, unhealthy, and therefore banned nematicides, such as aldicarb [4] and carbofuran [5]. Credible pathways and evidence summarization of relevant trends will follow herein.

Currently, intensive research with significant progress has been conducted to grasp the molecular basis of triggering, signaling, and expression of SAR (systemic acquired resistance) and ISR (induced systemic resistance). To provide an overview of related mechanisms and molecular players engaged in the relevant onset and expressions, a short glossary of key terms is presented. The terms are intended to briefly introduce the two domains detailed herein: boosting the use of PPN-resistant plant cultivars and optimizing the contribution of beneficial microbes in priming and immunizing plants especially against PPNs. Recent advances are highlighted herein via numerous examples, and relevant limitations and key gaps are identified as well. Therefore, this review focuses on a critical and updated synthesis to operationalize the optimization in these two domains. In this context, a strong conceptual distinction is also drawn between “holistic” and “mechanistic” approaches. This may help avoid some potential pitfalls in future research methodology that focuses only on mechanistic rather than a comprehensive approach. References were selected with molecular keywords closely related to these domains mostly in the last seven years unless a seminal finding or key principal was reported earlier. To initialize a unifying conceptual figure and improve coherence among the addressed domains, a short glossary of key terms is introduced.

### A Short Glossary

**PTI (Pattern-Triggered Immunity)**: The first line of induced immune response, known as Pathogen-Associated Molecular Pattern (PAMP)- or Microbe-Associated Molecular Pattern (MAMP)-, that is, PAMP- or MAMP-triggered immunity (PTI). It is activated when plant pattern recognition receptors (PRRs) on the cell surface detect conserved molecular signatures, e.g., PAMPs or MAMPs. Upon recognition, the plant triggers basal resistance, e.g., callose deposition for cell wall fortification, reactive oxygen species (ROS) production, Mitogen-Activated Protein Kinase (MAPK) signaling, and transcriptional changes.

**ETI (Effector-Triggered Immunity)**: The second, more potent line of defense. It is activated when resistance (R) proteins, typically nucleotide-binding, leucine-rich repeat (NLR) receptors, inside the plant cell directly/indirectly recognize specific pathogen, e.g., PPN effectors.

**ETS (Effector-Triggered Susceptibility)**: A state of enhanced susceptibility that occurs when a pathogen successfully overcomes PTI. Thus, PPN-effector proteins enable their establishment and reproduction on susceptible plants.

**SAR (systemic acquired resistance)** and **ISR (induced systemic resistance)**: The two main types of induced, whole-plant resistance mechanisms that protect plants against pathogens/pests (herein PPNs). They are activated by specific environmental/chemical stimuli or biological agents, “preconditioning or priming” the plant for a rapid and robust defense response. While **ISR** is thought to be acquired upon local induction by beneficial microbes, **SAR** is putatively acquired upon local induction by a pathogen, with emerging controversy. Yet, recent research has shown that the boundaries between these two pathways are often fluid (this is discussed herein).

**Priming the plant**: An adaptive mechanism to enhance a plant’s defense system. The plant is pre-conditioned for efficient defense against biotic/abiotic stresses. It allows the plant to respond more rapidly, strongly, or efficiently to future stresses (e.g., PPN infection), without the high energetic costs linked to constantly maintaining a fully activated defense state. It enforces the plant immunological memory, where an initial, low-level stimulus, i.e., ecological/chemical or biological agent (warning signal), prepares the plant for a more robust defense upon later, e.g., real PPN infection.

**Microbiome engineering**: The intended manipulation, design, and modifying of microbial communities (microbiomes) within a definite habitat usually to boost the positive ecosystem’s operation and/or stability. It uses engineering principles to attain the related outcomes.

## 2. The Holistic Approach of Plant–Soil–Microbiome Complex

**Figure 1:**
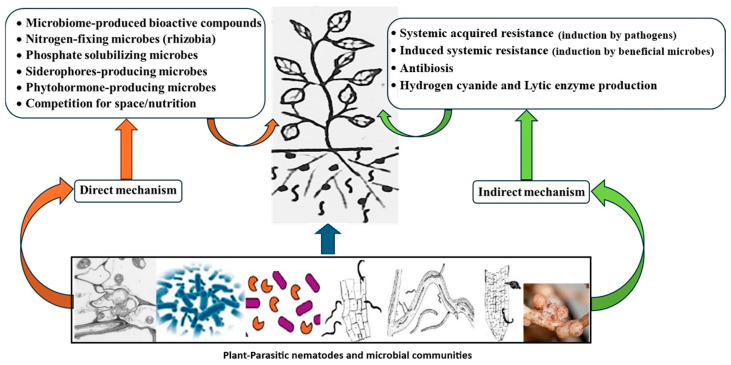
Diverse microbiome activities in the rhizosphere to boost plant growth and/or strengthen its molecular biology-based immunity response.

This system acts as an integrated whole; therefore, individual components cannot be fully recognized by studying them in isolation. That is because plant immunity/resistance emerges as a component within complex interactions of the entire system linked with the environment, not a single gene, signaling, or microbe. Strikingly, environmental factors, e.g., soil properties regarding soil nematodes, dynamically impact the entire community structure and performance. Edaphic factors can offer insight on modulating soil attributes to boost biocontrol and/or microbiome by favoring and maintaining certain settings. Mulching [6], soil texture and moisture [7], salinity [8], and pH [9] significantly (*p* < 0.05) modulated nematode population levels. Such factors exerted direct influence on the soil fauna and flora, but they also had indirect impact on hosts and enemies of soil nematodes [10]. Modifying orchard planting sites from “flatwoods” soil to sandy soil of the central ridge in Florida, USA, conserved biocontrol agents (BCAs). It enhanced entomopathogenic nematode (EPN) species richness (*p* = 0.001) and diversity (*p* = 0.01), reduced weevil herbivory (68%), and produced 85% more citrus fruit (*p* = 0.001) than those in flatwoods/native soil [11]. In another study, a new citriculture system that fertigated plants daily, thereby changing the soil properties, in order to combat citrus greening disease suppressed native and augmented EPNs there [10]. The new system increased soil pH and Mg content but reduced the electrical conductivity and content of K, Mn, and Fe. Consequently, the naturally occurring EPN *Steinernema diaprepesi* was five times less abundant in plots of citrus trees where these nematodes were more heavily encumbered by the phoretic bacterium *Paenibacillus* sp., which limits the foraging/biocontrol success of EPNs [10]. This context-dependent nature means that a beneficial microbe in one environment may not be as effective in another. Furthermore, the holistic approach considers diverse benefits of these microorganisms under specific settings. Collectively, their activities via direct and indirect mechanisms (Figure 1) to diminish stresses and enhance plant growth can raise plants’ ability to withstand pathogen/pest attacks for sustainable farming.

The approach also engages relevant microbiome engineering strategies, i.e., developing synthetic microbial communities for additive or synergistic microbial interactions. For instance, dual inoculation of mycorrhiza, *Rhizophagus irregularis* (Błaszk., Wubet, Renker & Buscot) Walker & Schüßler (Glomerales: Glomeraceae), and plant-growth-promoting rhizobacteria, PGPR, *Pseudomonas jessenii* Verhille, et al. (Pseudomonadales: Pseudomonadaceae) strain R62 and *Pseudomonas synxantha* (Ehrenberg) Holland strain R81) on tomato showed significant (*p* < 0.05) excess in plant growth and suppressed infection by *Meloidogyne incognita* (Kofoid & White) Chitwood (Tylenchida: Heteroderidae) [12]. Such a technique may delve into their mechanism too. This dual treatment manifested more activities of phenolics (28%) and defensive enzymes: peroxidase, POD (1.26-fold), polyphenol oxidase, PPO (1.35-fold), and superoxide dismutase (1.09-fold). Meanwhile, a significant decrease in malondialdehyde (1.63-fold) and hydrogen peroxide (1.30-fold) content was reported relative to the untreated inoculated check [12]. A combined treatment may use plant-beneficial microbe(s) with any other compatible input(s). The single treatments of *Trichoderma harzianum* Rifai (Hypocreales: Hypocreaceae) recorded 77.2% as an average total percentage of *M. incognita* reduction, but its combination with powdered leaves of basil raised it to 86.4% [13]. The holistic science of integrated pest management (IPM) also stresses systematic studies on the compatibility and optimization of concurrently implemented actions associated with at least two pests/pathogens. Because EPNs and/or their symbiotic bacteria suppressed infection by PPNs [14] and reduced populations of insect pests simultaneously under field conditions [6], Kepenekci et al. [15] stated that an underestimate of EPN application value sometimes occurred, as its value was based only on insect pest control.

Eventually, the interconnection/overlapping of the holistic and mechanistic approaches can occur simultaneously and/or sequentially over time, making their separation for boosting plant immunity against PPNs challenging, e.g., leverage plant-immune memory starts via defense priming of the targeted plant, but it may include potential transgenerational epigenetic inheritance of enhanced resistance, as will follow. Both approaches can use tools like clustered, regularly interspaced short palindromic repeat (CRISPR) and genetic editing, alongside techniques such as probiotics and metabolite manipulation within broader (environmental) or tighter (mechanistic) framing. Such tools/techniques and other agricultural actions to enrich disease-suppressive microbes are addressed herein, largely for PPN management.

## 3. The Mechanistic Approach for Combating Phytonematodes

The mechanistic approach focuses on grasping and targeting definite, detailed biological/physical processes of the host, the PPN, and their interaction at a molecular level. Thus, it contrasts with holistic methods by breaking down complex disease phenomena into their original components to initiate or develop PPN control strategies. Hence, the mechanistic approach can probe new disease resistance genes, their activation mechanisms, and signaling networks, which ultimately frames a more holistic grasping of plant defense to be exploited for pest control. Therefore, further optimization of plant resistance and the microbiome’s role against PPNs is addressed as follows.

### 3.1. Molecular Plant Mechanisms for Combating PPNs Engaging R-Genes

It is well established that plants possess several modes of action for protecting themselves [16]. Initially, recognition of specific PPN-molecule signatures induces innate immune reactions in the attacked plants. The common term for this ordinary innate or basal immune system is the primary layer or first line of defense against PPNs, the PTI [17]. It can detect microbe- (MAMPs) or nematode-associated molecular patterns (NAMPs) using pattern recognition receptors (PRRs) as special protein receptors (receptor-like kinases [RLKs] and receptor-like proteins) that can act to initiate this innate immunity, e.g., against root-knot nematodes (RKNs, *Meloidogyne* spp.) [18]. A striking example of an NAMP is ascaroside (Asc#18), which is secreted by PPNs: e.g., the cyst nematode (CN) *Heterodera glycines* Ichinohe (Tylenchida: Heteroderidae) [19]. On the other, the Arabidopsis leucine-rich repeat (LRR) receptor-like kinase NILR1 (nematode-induced LRR-RLK1) acts as the PRR for this ascaroside. Activating MAMP- (MTI) or NAMP-triggered immunity (NTI) by Ascaroside #18(ascr#18), induces distinct plant defenses [20]. In order to offer plant protection, according to Klessig et al. [20], NTI comprises consequent activation of mitogen-triggered protein kinases, salicylic acid- and jasmonic acid-interceded defense signaling pathways and defense gene operation. Although Ascr#18 is a member of a family of PPN signaling molecules, its exogenous application in low nanomolar/micromolar concentrations could offer protection against a wide spectrum of pests/pathogens. These latter include not only PPN species but also viruses, fungi, bacteria, and oomycetes [21]. For PPN control, the NTI results in generating reactive oxygen species (ROS) and secondary metabolites, cell death encompassing the PPN-migratory tunnel, and/or strengthening plant-cell walls [22]. These plant responses may slow down the early stages of nematode infection. They contribute to tangible defenses in non-host plants. However, PPNs can defeat NTI by secreting effector proteins called effector-triggered suppression (ETS) to impede the innate immune reactions in nematode-susceptible plants. Effectors such as Ha18764, RHA1B, GrVAP1, and GrCEP12 are formed by the nematodes in their esophageal glands and transferred by PPN stylet into the plant roots regarding soil PPNs [17]. Having suppressed the basal immune responses, endoparasitic sedentary nematodes become able to establish feeding sites. These sites, represented by giant cells for RKNs and syncytia for CNs, are essential for RKNs and CNs to develop and reproduce on their susceptible hosts. These susceptible plants initially show a weak upregulated defense, which is then suppressed by PPNs to ease the setting of a functional feeding site for perfect parasitism, known as ETS. Unlike PPN-resistant plants, gene upregulation focuses on rapidly activating strong, localized defense responses that result in the containment and death of the nematode, known as ETI. Thus, PPN-plant defenses are the main reason why some plants are more resistant than others. Importantly, resistance levels vary based on a plant’s genetic ability to recognize PPNs promptly and its vigor in using physical/chemical barriers against them. Factors for differences in plant resistance engage genetic recognition (*R*-Genes), physical and structural barriers, plant-secondary metabolites, and the ability of PPN populations to co-evolve and become breaking-resistance pathotypes [23,24].

Credible pathways to using related and reformed plans in creating plants usefully resistant to PPNs have been materializing. Root exudates belonging to the group benzoxazinoids are primarily released in many cereals, e.g., the rye plant, and exhibit toxicity against different PPN species. Cultivars of rye with high concentrations of methoxy-substituted benzoxazinoids in their roots were recommended to incorporate in the soil as green manure to protect against RKNs [25]. Such practices are especially important, as many cultivars of solanaceous and cucurbitaceous crops are highly susceptible to RKNs [26]. Molecular reactions triggered by such exudates that act as repellents, attractants, and toxic compounds against PPNs were documented to modulate the gene expression, including the large family of FMRFamide-like peptide (FLP) genes in nematodes [25]. These genes are responsible for several functions in PPN reproduction and thus have a vital role in the chemotaxis of PPNs. Furthermore, as many vegetable cultivars are less resistant/more susceptible to various nematode species in PPN-infested fields, it is quite common to rotate them with more resistant or poor-host ones. Small grains (e.g., wheat, barley, and oats) are mostly effective non-hosts for many PPNs (e.g., RKN and cyst nematode, SN) and may be rotated with susceptible vegetable cultivars. Interestingly, rotation sequences of grafted tomato-melon-pepper-watermelon on resistant rootstocks could revert the virulent *Meloidogyne incognita* populations on the *Mi1.2* gene and consequently minimized yield losses compared to ungrafted plants [27]. A soybean cultivar Nongqing 28 with stable resistance to the soybean cyst nematode (SCN), *H. glycines* race 3, and superior agronomic traits was recently released after 5-year field experiments [28]. The resistance was moved from the parent line PI 437654. The RNAseq analysis showed that its resistance is involved in pathogen perception and defense activation, such as ROS burst, calcium-mediated defense signaling, hormonal signaling, and phenyl-propanoid biosynthesis. Other SCN-resistant cultivars, such as KangXian, Nongqingdou, and Heinong 531, have been developed and released in the market in Heilongjiang, China [28,29]. Another approach for developing PPN plant resistance is based on combining grafting with breeding. Following *Meloidogyne incognita* infection, Li et al. [29] found that the tobacco grafted progeny GHF1 [F1 progeny of tobacco grafts ‘G278 (resistant rootstock) + Honghua Dajinyuan (HD, susceptible scion)] showed notably enhanced resistance compared to its scion HD. The obtained resistance was characterized by raised chlorophyll levels, increased activity of phenylpropanoid metabolic enzymes and disease-related proteins, reduced membrane lipid peroxidation, and stable antioxidant enzyme levels. The authors speculated that the employed technique has induced widespread alterations in specific DNA methylation patterns at particular loci, particularly in the scion, and these changes in DNA methylation at specific loci were inherited by progeny via sexual reproduction.

### 3.2. Current Problems Linked to Using R-Genes with Possible Genetic Solutions

As the spread of PPNs increases and their related losses are worse, it becomes clearer that the number of nematode-resistant plant species/cultivars is insufficient, especially in key crops. Moreover, an individual *R*-gene in the cluster may be employed for resistance, but multi-gene background is typical for plant *R*-genes [20]. For example, *Mi-1* contains a limited gene cohort with seven homologous copies jointly grouped on the small arm of chromosome 6 in the tomato-genetic constitution with RKN resistance [30]. Nonetheless, various *Mi* genes exist that differ from *Mi-1* in specificity, functional characteristics, and genetic locations [31,32]. In RKN-resistant tomatoes, out of ten *R* genes dedicated for resistance, only seven genes can function at high temperatures, i.e., above 32 °C [33]. Recently, Devran et al. [33] found a tomato line, *MT12*, that can offer resistance to *M. incognita* at 32 °C soil temperature. Mapping RRKN1 to chromosome 6 using Kompetitive Allele Specific PCR markers provides a genetic resource and opens novel avenues for breeding tomato varieties with stable resistance (at ≤32 °C soil temperature [33]) under such conditions. On the other hand, a slow decline in the effectiveness of *R*-genes could sometimes be recorded. A striking example is the present issue in incorporating resistance derived from plant introduction accession 88788 into almost all *H. glycines*-resistant soybean varieties planted in the USA. Although this CN is a key pest of the soybean plant there, its relevant resistance—encoded by a high copy number of the rhg1-b allele—has already begun to decline. Therefore, incorporating *Bacillus thuringiensis* (*Bt*) Berliner (Bacillales: Bacillaceae) delta-endotoxin (Cry14Ab) into soybean plant could boost H. glycines resistance in the genetically engineered soybean [34]. The latter authors demonstrated that *Bt* delta-endotoxin, Cry14Ab, controls SCN in transgenic soybean in a similar mechanism to their experiments on *Caenorhabditis elegans*. This mechanism involved damaging the nematode intestine, similar to the mechanism of Cry proteins used to control insects. Thus, plants expressing Cry14Ab showed a significant reduction in cyst numbers compared to control plants 30 days after infestation. In addition, their field trials showed a reduction in SCN egg counts compared with control plants, confirming that this protein has excellent potential to control PPNs in soybean [34]. It is still desirable to identify more resistance sources with broad-spectrum resistance to *H. glycines* and favorable agronomic traits. So, by assessing molecular marker haplotypes at the rhg1 and Rhg4 loci and testing resistance against multiple *H. glycines* races, 12 soybean cultivars were found to exhibit Peking-type resistance, effective against multiple nematode races [35]. Their detection offers valuable genetic resources for breeding *H. glycines*-resistant soybeans. Yet, further related investigations are needed, as there are two types of resistant soybean sources that are widely used against SCN. These include Peking-type soybean, whose resistance requires both the rhg1-a and Rhg4 alleles, and PI 88788-type soybean, whose resistance requires only the rhg1-b allele.

Another problem with using *R*-Genes is linked to the gene construct itself. Tomato plants with dual genes (PjCHI-1 and CeCPI) and synthetic promoters produced transgenic lines that possess better resistance to RKN infection and multiplication than corresponding transgenic plants with a single gene [36]. Moreover, most plant resistance genes utilized so far are operating against only the endoparasitic sedentary nematodes [20]. So, other categories of key PPN species should be examined to develop their resistant plant cultivars/varieties. This would also include differentiating those cultivars/varieties with resistant dual genes versus single genes. For instance, migratory endoparasitic nematodes such as the rice root nematode *Hirschmanniella oryzae* can cause significant yield losses. While *H. oryzae* does not induce a feeding structure, it can either completely enter the root, making damaging tunnels, or just embed its head into the root cortex to feed on root nutrients. Considerable rice losses due to *H. oryzae* were 25% in infested fields [37]. Chorismate mutase was found to be secreted by *H. oryzae* to inhibit SA synthesis in order to suppress the defense of rice plants against *H. oryzae* [38].

Resistance-breaking pathotypes have been widely recorded [18,32,39,40]. For such RKN pathotypes, their populations could be sourced from populations recovered from resistant tomato-cultivated fields, natural virulent populations (not from resistant tomato fields), and avirulent populations selected for virulence in the laboratory [41]. It is apparent that the genetic events leading to the acquisition of these pathotypes or virulent populations against the *Mi*-gene differ according to their source, i.e., selected versus natural populations. Moreover, Molinari [42] speculated that selection pressure for virulent populations could also acquire another function that enabled the nematodes to escape the host reaction, e.g., by boosting activities of antioxidant enzymes. Unfortunately, such virulent populations are diffusing [18]. Other factors may contribute to their spreading such as ecological complexes engaging changes in populations of multiple PPN species [43] and temperature [18]. Conversely, a few techniques can help to develop plants resistant to such virulent populations. It would be wise to utilize diverse resistance genes such as major *R*-genes and minor genes and incorporate them within IPM to slow down the evolution of virulent PPN populations, which can evade single-gene resistance [44,45]. Moreover, genomic devices are being directed to accelerate the discovery and activation of new genes for sustainable crop protection. They would preferably be used to manage the host plant–nematode pest co-evolution for long-term success against such virulent populations. Moreover, Ibrahim et al. [46] discussed a few molecular approaches to grasp plant-nematode interaction mechanisms and consequently improve plant resistance against PPNs, e.g., genome sequencing technologies, small interfering RNA techniques (RNAi), and targeted genome editing.

In addition to the above-mentioned efforts to advance PPN-resistant cultivars, conceptual revision with critical synthesis to operationalize “the optimization” is still direly needed. Basically, wild relatives of key crops are highly necessary for breeding to develop PPN-resistant plant genotypes. The current intensive breeding has often resulted in a narrow genetic base, causing domesticated varieties to lose natural resistance genes, e.g., via the aforementioned resistance-breaking pathotypes. Hence, these wild relatives will widen the related genetic pool. They can serve as the main source of new genetic diversity for developing resistant, durable, and high-performing genotypes/cultivars. Breeding of chickpea for PPN resistance has many challenges that originate from the narrow genetic diversity. To overcome these challenges, Zwart et al. [47] stressed that harnessing molecular techniques, e.g., molecular marker-based resources, next-generation sequencing-based resources, and genome-assisted breeding, to leverage the relevant wild types is promising. For instance, chickpea breeding via molecular marker-based resources together with robust/accurate phenotyping to detect marker–trait associations can offer three merits, i.e., help the indirect selection of PPN resistance, facilitate pyramiding of resistance genes from several resistant or moderately resistant sources to provide cultivars with durable resistance, and merge the resistance with multiple biotic stresses. On the other hand, Gohar et al. [48] integrated a classical approach into single nucleotide polymorphisms (SNPs) as molecular markers for host susceptibility designation of sugar beet to *M. incognita* infection. They effectively rated different sugar beet genotypes as tolerant, susceptible, or resistant to the nematode. The multiple parameters-based rating revealed significant differences among beet genotypes regarding disease severity, yield production, and quality traits. The screened high-yield genotypes with tolerance/resistance to *M. incognita* can help sugar beet breeders generate novel commercially desirable genotypes. In addition, SNP markers can help efficiently identify resistant and susceptible genotypes. Similarly, these lines of thinking may apply especially to other key crops. In other words, molecular markers and genomic data as more accurate and reliable tools should be included alongside traditional methods or even replace them in screening for and development of PPN plant resistance.

Conflicts in the results of plant breeding for PPN resistance frequently arise from the complex nature of the plant–PPN interaction as influenced by genetic, ecological, and behavioral factors. They may stem from variability in nematode populations (e.g., pathotype variability), genetic and physiological factors (polygenic nature of resistance), ecological factors, and technical and methodological differences. The latter can help reduce all these problems. As it is almost impossible for relevant researchers to act united, they need to use standardized procedures that allow future reviews to be analytical and possibly build on them. If so, development of morphological modifications, e.g., syncytium induced by CN, and giant cells induced by RKN are crucial for their survival. However, many resistance genes preclude growth of these feeding sites or induce localized necrosis, thus restricting female development and reproduction. Moreover, defense pathways involving transcription factors (RAP2.6, WRKY33), hormonal crosstalk (JA, SA, ET, ABA), and ROS-mediated hypersensitive responses further disrupt feeding site establishment. Collectively, such actionable pathways can guide resistance breeding plans to disrupt nematode feeding structures and ensure durable control [49]. The procedures should use sound statistical analyses and interpretations of results. As breeding for resistance and the microbiome are often more sensitive to biotic/abiotic factors than chemical pesticides, three different statistical levels (*p* ≤ 0.10, 0.05, and 0.01) of probability may be used. The levels have been proposed for non-molecular breeding schemes only [50] but may better be used for integration with molecular ones. They may enable, for example, distinguishing slightly resistant, moderately resistant, and highly resistant plants for the three levels. For both accurate and precise quantification of PPN and related microbes, it is better to use the confidence interval of the mean value than the standard deviation, which is commonly used in many relevant studies. Precision level can indicate consistency or low variability among such sensitive experimental replicates, but it also confirms whether results are reproducible. Moreover, this level can help in determining the optimum dose/concentration of a treatment to avoid erratic results. For instance, application of the phytohormone salicylic acid (SA) is generally known to strengthen plant immunity against nematode attack [51]. However, its dosage, which was lower than optimal, could not perform its assigned role, i.e., adversely affecting RKN infection to the plant hosts. On the contrary, fairly higher ones were toxic. Meanwhile, other solanaceous species were stimulated differently against RKN by optimal SA rates [52].

**Figure 2:**
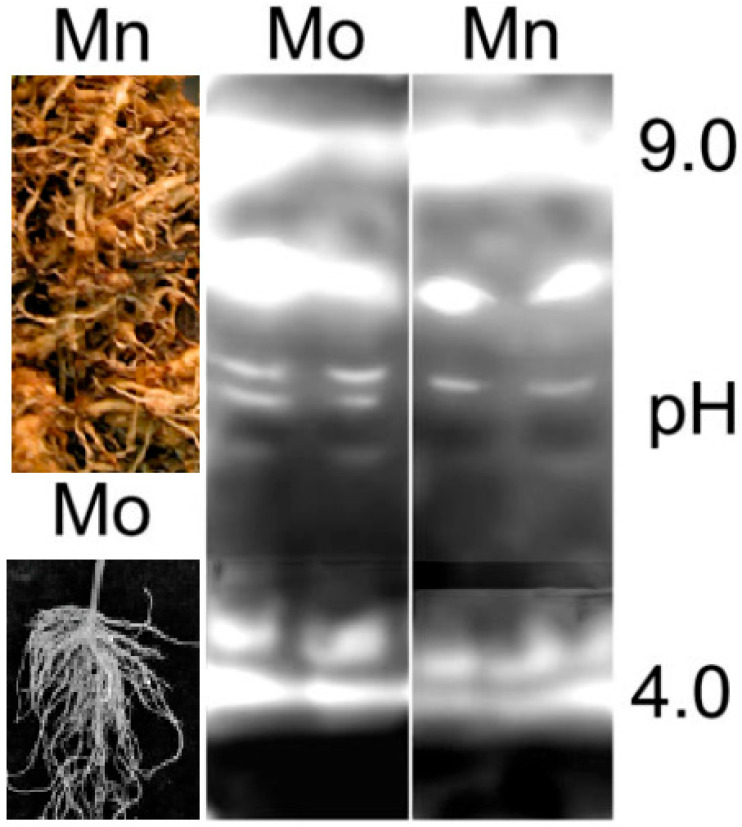
Isoelectrofocusing for bands of catalase activity in tomato root extracts of the resistant cv. Motelle (Mo) and susceptible cv. Moneymaker (Mn). Their infected plant roots are found on the left [53].

A tantalizing possibility is still aiming to save costs, time, and efforts via pushing forward molecular techniques of pathogenesis-related proteins. When two near-isogenic cultivars (Motelle and Moneymaker), differing mainly in the presence and absence, respectively, of the gene *Mi-1.2* (responsible for resisting RKN), were used, a significant difference was noted in the catalase (CAT) activity of roots due to *M. incognita* infestation [53]. This difference is directly dependent on a specific action of the gene challenged by nematode attack. Moreover, CAT activities (expressed as units × mg^−1^ protein) of root extracts from seedlings of the *M. incognita*-resistant cv. Motelle and susceptible cv. Moneymaker averaged 34.4 and 18.9 in the two uninfected tomato cultivars, respectively (Figure 2). As CAT inhibition is likely to be associated with the oxidative burst producing H_2_O_2_ in incompatible tomato–RKN interactions, this biochemical event may also be expressed in resistance conferred by genes different from the most well known, *Mi-1.2* [53]. Importantly, using the content in catalase in readily available materials, such as seeds, to screen germplasm groups, without the additional tasks of growing plants and inoculating nematodes, would be of even greater interest. Admittedly, more investigations with proper tools and sophisticated techniques should be directed to strengthen plant immunity/resistance.

### 3.3. Beneficial Roles of Plant-Associated Microorganisms

**Figure 3:**
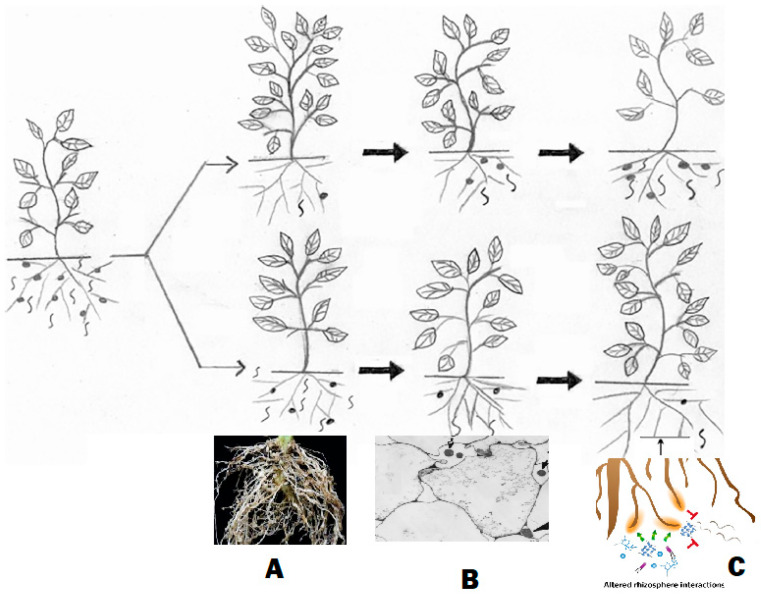
Effect of a chemical nematicide (upper) and a microbiome community (lower) on root-knot nematodes (RKNs) on a susceptible plant. The chemical has a quick and notable impact on reducing the RKN population, but a few nematodes can escape its effect and reproduce to reach a damaging level. On the contrary, the microbiome [mycelium of biocontrol fungi covers the root for protection (**A**), hyphae of arbuscular mycorrhizal fungi penetrate the root into the epidermis and outer cortex to strengthen their cells via deposition of newly formed barriers, e.g., callose (**B**), and plant growth-promoting rhizobacteria fortify plant immune responses (**C**)] can act continuously to keep the nematode below the economic threshold level.

Having identified the resistance genes-related issues and defects, it is necessary to search for a complementary framework to enhance plant immunity against PPNs. This does not negate the need for further research to solve relevant issues of the aforementioned plant–nematode interactions. In addition, the fruitful role of the microbiome in plant immunity requires close scrutiny to identify its best exploitation. Strikingly, a standard type of accrued soil microbiome competitive tasks is assumed to not only function against PPNs. It can frequently act against a broad range of soil-borne diseases in addition to abiotic stresses ([54,55], Figure 3). Therefore, many firms do not sell numerous of their products containing BCAs that can indirectly combat PPNs, e.g., formulated bacteria or fungi, as bionematicides, such beneficial soil-borne microorganisms reinforce plant immunity in different ways. Hence, they are preferably addressed herein under the following subtitles.

*A*. *Categories of Microbiomes and their molecular activation of Plant immunity*

Although BCAs of PPNs comprise a wide array of beneficial soil-borne micro-organisms, only definite and related categories can activate plant immunity. For example, different species of trapping fungi and predatory nematodes [56] are used against PPNs without activating plant immunity; both groups are considered predacious microorganisms. In contrast, while predatory nematodes move for their prey, nematode-parasitic *Pasteuria* spp. initiate their life cycles when the endospores passively get attached to the outer cuticle layer of their specific host nematode population/species, as the PPN individuals move through the soil [57]. Once adhered, the bacterial endospore germinates, producing a germ tube, which penetrates into the host, to infect the nematode body by multiplying bacterial cells. Interestingly, it was postulated that such microorganisms attaching to the infective stages of endoparasitic nematodes activate defense mechanisms in plants after they enter the roots [58]. Apart from such putative effects, BCAs that can colonize roots and stimulate plant immunity to PPNs could generally be divided into three main and widespread categories. These are arbuscular mycorrhizal fungi (AMF), PGPR, and biological control fungi (BCF) [59]. As most PPN species are soil parasites, plant roots colonized by useful microorganisms of these categories have been recorded to generate systemic acquired resistance (SAR)-like responses against the nematodes [52]. For instance, AMF are obligate root symbionts, but they behave like pathogenic fungi at first and begin with secreting defense suppressors to realize thriving colonization on roots. Thus, AMF are faced by SA-regulated defenses, which show steady likeness between receptors and processes engaged in symbiosis and immunity molecular signaling [60,61]. Hence, SA-dependent *PR-1* gene was upregulated only 3 days after AMF treatment, suggesting that the inoculated tomato plants had first recognized AMF as a pathogen [62]. Thereafter, AMF could repress this reaction as a routine system so that AMF realized good colonization. Eventually, mycorrhiza-colonized plants are primed (pre-conditioned for efficient activation of plant defenses) against different pathogens like PPNs. So, the response of the plants primed to a pathogen attack occurs faster and/or stronger compared to plants not previously exposed to the priming stimulus. This status, named mycorrhiza-induced resistance (MIR), is well documented for prompt and strong reaction to varying biotic attacks [52,63,64]. Plant-growth promotion is often noted due to enhanced acquisition of mineral nutrients through the arbuscular mychorrhizal (AM) fungal hyphal network. Furthermore, changes in the root exudate patterns repel nematodes and induce changes in the soil microbial community, possibly attracting antagonists of pathogens. For PPNs, MIR could prime or immunize plants especially via upregulation of PR- (pathogenesis-related) and ACO- (1-aminocyclopropane-1-carboxylate oxidase) genes as well [62]. Such priming and immunizing of plants against RKNs was based on SAR [51].

Interestingly, effectors of many microorganisms in the rhizosphere not only can manipulate the structure/function of the host plant cells to set infection as virulence factors but may also trigger a defense response as avirulence factors in the plant host [65,66]. Some effector proteins of beneficial microorganisms have been experimentally analyzed during their interactions with plant systems. Their interactions have been harnessed to fortify plant immune system. For PPNs, Könker et al. [67] used *Pochonia chlamydosporia* (Goddard) Zare & Gams (Hypocreales: Clavicipitaceae) to synergistically reinforce systemic plant defense reaction of *Phacelia tanacetifolia* against *Meloidogyne hapla* Chitwood (Tylenchida: Heteroderidae). Transcriptome and metabolome analysis of plant leaves revealed that the metabolome was quite stable except for the first 2 days post-inoculation (DPI). Comparing *P. chlamydosporia* singly with *M. hapla* + *P. chlamydosporia* treatment manifested larger impacts after 6 compared to 2 DPI, aligning with the later root infestation by *P. chlamydosporia* relative to *M. hapla*. Hence, *M. hapla* had a stronger effect on the shoot transcriptome at the early time point of 2 DPI than 6 DPI, since 548 transcripts revealed increased abundance after 2 days but only 239 transcripts did so after 6 days. In contrast, *P. chlamydosporia*-treatment effects become more apparent with increasing duration after treatment. Shifts in transcripts and metabolites were higher in the combined treatment relative to the single inoculum, which supports the conclusion that *P. chlamydosporia* induces plant defense in a distinguished and beneficial manner when combined with *M. hapla*. The largest portion of differentially expressed genes occurred with *M. hapla* + *P. chlamydosporia*, namely 392 decreased and 216 increased transcripts at 2 DPI and 285 decreased and 259 increased transcripts at 6 DPI. This was reflected thereafter by a 24.3% reduction in the *M. hapla* population when combined with the fungus compared to the treatment *M. hapla* alone at 28 DPI. Additionally, the numbers of *M. hapla* eggs extracted from plant roots and quantified were 247 ± 44 and 58 ±10 eggs/g root dry weight in *M. hapla* alone and *M. hapla* + *P. chlamydosporia*, respectively [67]. Moreover, these results proposed that *P. chlamydosporia* application against *M. hapla* can be more effective via backing the underlying tritrophic (microbiome–nematode–plant) interactions with specific additives, e.g., phytohormones or amino acids. Ghahremani et al. [68] proved that isolates of *P. chlamydosporia* induced plant-dependent systemic resistance to *M. incognita* in tomato. The fungal isolate M10.43.21 reduced infection (32–43%), reproduction (44–59%), and female fecundity (14.7–27.6%), while another isolate M10.55.6 consistently decreased nematode reproduction (35–47.5%). Interestingly, the isolate M10.43.21 induced the expression of the SA pathway (*PR-1* gene) in tomato roots 7 days after being inoculated with the fungal isolate and just after *M. incognita* inoculation and at 7 and 42 days after nematode inoculation [68]. The jasmonate signaling pathway (*Lox D* gene) was also upregulated at 7 days after *M. incognita* inoculation. Other studies on PPNs revealed that compounds that decreased SA and/or ROS levels increased PPN infection severity, while those that reduced phytohormones jasmonic acid (JA)/ethylene (ET) levels did not generally affect infection rates [51]. For example, the effect of overnight root dipping in a ROS scavenger solution on tomato plants inoculated with *M. incognita* showed marked changes relative to untreated inoculated plants. The average plant weights of shoots in grams and number of egg masses per gram of root fresh weight were significantly (*p* ≤ 0.05) different as nematodes were allowed to complete their life cycle: 9 and 53 in untreated compared to 12.3 and 70 in treated plants, respectively [51]. That is because ROS scavengers are so important for resisting PPN, but their impact is a double-edged blade. The key role of such scavengers is to detoxify damaging ROS during stress on the plant. However, over-scavenging or disrupting the delicate ROS balance can debilitate defense signaling. Consequently, these scavengers would retard a strong immune response against pathogens/environmental stresses. In other words, the right amount of ROS is required for the signaling of plant defense, but excessive scavenging can lessen defense signals reflected by upregulation of related enzymes and reduced resistance, while balanced ROS promotes overall plant health and stress tolerance. In another study [69], soil pre-treatment with *T. harzianum* strains ITEM 908 (T908) and T908-5 decreased susceptibility of tomato to *M. incognita*, as assessed by restriction in nematode reproduction and development [69]. The effect of *T. harzianum* treatments on plant defense was detected by monitoring the expression of the genes *PR-1*/*PR-5* and *JERF3*/*ACO*, markers of the SA- and JA/ET-dependent signaling pathways, respectively. The compatible nematode–plant interaction in the absence of fungi caused a significant (*p* ≤ 0.05) suppression of *PR-1*, *PR-5*, and *ACO* gene expressions, either locally or systemically [64]. Notably, some *Trichoderma* spp. can antagonize PPNs and other plant pests, offering them as promising multifunctional BCAs [55,70].

Such microbiomes are also added within bio-fertilizer formulations that may include many types of composts. Recently, a food waste compost (FWC1) mixed with soil increased plant biomass by 30% compared to untreated plants [71]. Moreover, when these tomato plants were inoculated with *M. incognita*, treated roots contained about 50% less sedentary forms of nematodes and a lower reproduction rate of the parasites than untreated plants. Although the effect of FWC1 as a defense activator was similar to that of other microbiome-generating commercial formulations, the compost was found to be the best fertilizer in both un- and inoculated plants. Spread root colonization by AMF was noted after treatments with FWC1. In addition, FWC1 water extracts did not show any toxic impact on living *M. incognita*-juveniles. Quantitatively, expression of the marker gene of immune response PR4b was 3–5-fold higher in the roots of inoculated plants treated with FWC1 relative to untreated plants. Thus, the authors [71] concluded that FWC1 primed plants against RKNs.

Moreover, PGPR can suppress diseases either directly via synthesizing pathogen-antagonizing compounds or indirectly through triggering plant immune responses. Clearly, their general merit is to contribute to stimulating plant growth especially by inhibiting plant-pathogenic organisms. Jiao et al. [72] reviewed diverse mechanisms of rhizobacteria for suppressing diseases. One or several mechanisms in combination can be associated with specific host plants. Their well-known direct mechanisms in biocontrol scenarios are suppressing pathogens via generating different anti-pathogen compounds, e.g., antibiotics, bacteriocins metabolites, antimicrobial peptides, toxins, and enzymes. They noted that volatile organic compounds generated by PGPR play a significant role in enhancing plant growth and provoking induced systemic resistance (ISR). Further, recent studies found a positive correlation between photosynthesis boosted by PGPR and host plant resistance level; photosynthesis offers materials and energy for plant-immunity and affects defense-related signaling pathways [73]. Photosynthesis organelles reinforced by PGPR inoculation are key centers for defense signal biosynthesis and transmission. As PGPR can raise soluble sugar levels and alternate sugar type and distribution, these shifts back plant growth and function as secondary messengers under stressed conditions. Thus, carbohydrate metabolism modifications induced by PGPR also played a key role in enhancing plant immunity [73]. Hence, it was stressed that coordination of these plant defense mechanisms holds potential for climate change-resilient agriculture [74]. Other bacterial species of genera like *Pseudomonas* and *Bacillus* are recorded as BCAs that trigger systemic resistance too in the plants’ rhizosphere [75]. These perspective articles [72,73,74,75] reviewed molecular activation of plant immunity via beneficial microbiome with additional examples.

Practically, consolidated usage of PGPR and other bio-nematicides/chemicals has been commercially and experimentally successful. The plant activator acibenzolar-S-methyl (ASM) showed better persistence behavior in the tomato rhizosphere as well as more root uptake systemic translocation ability after its combination with certain *Bacillus* PGPR strains [76]. Furthermore, combined usage of the mutualistic endophyte *Fusarium oxysporum* Schlecht. emend. Snyder & Hansen (Hypocreales: Nectriaceae) strain 162 and *Bacillus firmus* Bredemann & Werner (Bacillales: Bacillaceae) was the most effective treatment, among other combinations, in controlling *Radopholus similis* (Cobb) Thorne (Tylenchida: Pratylenchidae) on banana (86.2% reduction in nematode population density), followed by *B. firmus* alone (63.7%). Mendoza and Sikora [77] attributed such high efficacy to the compatibility of the two BCAs and the capacity of *F. oxysporum* to colonize banana roots. In addition, they attributed the better *R. similis* control in banana to the combined usage of antagonists with different modes of action. Definitely, the combination enabled targeting different *R. similis* stages during the infection process [77]. Additionally, various microbial groups contained in a commercial formulation (Myco) were more effective in limiting RKN damage than a formulation containing the sole AMF [2]. For explanatory reconciliation, the exact effect of such beneficial biologicals on PPNs may depend on the involved species/strains of both the hosts and the microbes as well as on the related type of mechanism, compatibility of the engaged biologicals, methods, and rates/concentrations of the inoculants [2,78,79].

*B*. *Unravelling Molecular Complexities in Microbiome–Plant Immunity Interaction*

Trials are still ongoing to aid in unraveling molecular complexities in microbiome–plant immunity interaction. Five tomato cultivars susceptible to RKN were treated prior to the nematode inoculation with a set of chemicals closely related to hormones/processes of plant defense. The chemicals comprised inhibitors of SA- or JA-mediated defense pathways, hormone generators, compounds that interfere with calcium-mediated metabolism, ROS generators and scavengers, and inhibitors of ROS generation [51]. Before treatments, it was found that SA-responsive genes tested were downregulated in response to infection by *Meloidogyne incognita*. Generally, compounds that reduced SA and/or ROS levels enhanced *M. incognita* infection severity, but those that minimized JA/ET levels did not affect infection rates. Such reactions demonstrated SAR where the induced resistance is specifically mediated by the accumulation of SA and subsequent expression of *PR* genes [51,80]. The latter authors stated that SAR is activated by the attack of biotrophs, such as PPNs, or by treatment with chemical or natural materials. Parallel to this concept, the effect of *M. incognita* attack on the performance of the defense enzymes endoglucanase and endochitinase, encoded by *PR-2* and *PR-3*, respectively, changed relying on the tested DPI with RKN-second stage juveniles (J2s) [51]. Both enzymes were inhibited in inoculated plants compared to healthy controls at 5 DPI. Moreover, the genes encoding glutathione peroxidase and CAT, as components of the plant-antioxidant system, were highly overexpressed. In the infected roots, the activity of superoxide dismutase, CAT, and ascorbate peroxidase, as antioxidant enzymes, was elevated too. Within the context of SAR against biotic stresses, adding ROS generators could encourage resistance against *M. incognita*, but materials that silenced calcium signaling boosted infection symptoms. Such results [1] confirmed that SA-mediated plant immune reactions are consistently inhibited in the early stages of *M. incognita* infection to the susceptible cultivars. The inhibition was linked to the stimulation of the ROS-scavenging order. JA-responsive genes were unaffected by the RKN infection [51]. This may indirectly agree with ISR, as JA-responsive genes are mostly induced by the necrotrophic pathogens [81]. Yet, some studies stated that ISR can be induced not only by beneficial microbes like AMF and PGPR but also via treating with chemical/natural compounds to attack pathogens [80,81].

On the other hand, the stable recognition that enzymes such as CAT, phenylalanine ammonia-lyase (PAL), PPO, superoxide dismutase, POD, proteinase, b-1,3-glucanase ascorbate, chitinase, and lipoxygenase participate in the plant resistance mechanism by generating related chemicals as phytoalexins and phenolic compounds [75] is being stretched by further mechanisms and additional bacteria. Rhizobia are basically known for their mutualistic relation with legumes where the bacteria offer a good service by fixing atmospheric nitrogen. Yet, another economic benefit of rhizobia has been recently reported. Rhizobial invasion of host roots also triggered an elevated systemic resistance in the host plant [82]. Specific structural features of rhizobial cells are related with evading MTI, e.g., rhizobia do not possess the flg22 epitope (a well characterized MAMP). Moreover, the classical view of symbiosis evolution indicates that a successful interaction relies on the rhizobial ability to suppress the host innate immunity for a short duration. Thereafter, during the autoregulation of nodulation (AON) system, CLE peptides (small, plant-specific signaling molecules, peptide hormones) are produced in the roots. Hence, it is speculated that the AON system can be related with the systemic increase in the defense response (ISR-like responses induced by rhizobia). This is probably mediated by a primed state involving a hormonal crosstalk and epigenetic modifications [83]. This related mechanism needs to be further explored as it contributes to plant health by inhibiting a broad range of pathogens. The authors [82] stressed that molecular events mediating these responses are still not fully understood and could involve a mechanism that differs from that described for other PGPR. Very recently, field results revealed that the rhizobia impact on pests, virus, and pea yield exceeded that of nitrogen fertilizer [83]. In other words, rhizobia aided plant growth largely due to reduced pest levels; thus, their benefits extended beyond nitrogen fixation.

Fungal effectors can partly share in unraveling the interaction complexities. For better perception, Table 1 shows a few of these effector proteins in fungus–plant interactions, their families, related fungal species, and functions in interaction with plants. Such candidates of avirulence factors, both canonical and non-canonical, may be employed by beneficial fungi against definite pathogen species. To simplify, gene expression data indicate that considerable overlap exists between the plant-defense response induced by microbe-associated molecular patterns (MAMPs), effectors, and endogenous elicitors so that avirulence factors can also be redefined as virulence factors [84,85]. However, these and other similar candidates present in further interactions could be broad-host range candidates [85]. Therefore, future investigations are needed to determine which candidates remain specific to a pathogen species versus non-specific ones. Effector identification particularly in fungal–nematode–plant interactions is an understudied theme of investigation.

Tackling families of transcription factors (TFs) can also give valuable insights into the genetic and transcriptional dynamics associated with PPN invasion and development. Domínguez-Figueroa [94] recorded that current research on plant–PPN interactions has disclosed nearly 50% of all plant transcription factors (TFs) to be differentially expressed during infection. This percentage is specifically allocated to coordinate the development of specialized feeding sites like syncytium (for CN) and galls and giant cells (for RKN). Emerging patterns show that these TFs act as central pivots, integrating defense signaling with the massive cellular reprogramming required for PPN parasitism. Generally, TFs are manipulated by PPNs or utilized by plants via five emerging patterns. The most common one is redifferentiation of feeding sites where PPN effectors directly target host TFs to transform normal root cells into specialized feeding structures, such as giant cells or syncytia. Targeted TF families, the second pattern, may be grouped into three families. These are MYB and WRKY (mainly engaged in balancing growth reprogramming with the activation of plant immune signaling pathways, ERF (Ethylene Response Factors) that modulate ethylene-related defenses and cell wall modifications, and ARF (Auxin Response Factors) and LBD (LOB-domain) that are necessary for the auxin-driven organogenesis required to build feeding sites. In hormonal cross-talk regulation, the third pattern, TFs mediate the balance between SA and JA pathways. For example, the atypical TF DEL1 has been found to suppress SA accumulation in galls to prevent host resistance [94,95]. MicroRNA-TF regulatory modules, the fourth pattern, has a key role in post-transcriptionally silencing TFs to ease PPN infection. Notable modules include miR396/GRF in soybean [96]. In effector-driven interference, the fifth pattern, PPNs secrete “effector” proteins to inhibit TF DNA-binding domains or alter their activity to prevent the expression of defense-related genes [95].

Thus, plant TFs regulate the expression of instructions contained in the plant’s genes in response to PPN attack and growth (Table 2). Different comparative gene expression analyses are progressing with functional reviews of plant TFs and their relevance in the plant–PPN interaction [94]. Interestingly, such differential expression may even be materialized by the same host plant, e.g., *Oryza sativa* L. (Poales: Poaceae) infected by two different PPN species, i.e., *Meloidogyne graminicola* Golden & Brichfield (Tylenchida: Heteroderidae) and *Hirschmanniella oryzae* (Van Breda de Haan) Luc & Goodey (Tylenchida: Pratylenchidae) (Table 2). In another study, analysis of rice growth and agronomic traits of two independent mutants showed that there are no obvious differences between wild-type plants and two mutants. These findings suggest that susceptible (*S*) gene rice copper metallochaperone heavy metal-associated plant protein 04 (*OsHPP04*) may be an *S* gene as a negative regulator of host immunity. Thus, genetic modification of *S* genes through the CRISPR/Cas9 technology was used as a powerful tool to generate PPN-resistant plants. Thus, breeding for *M. graminicola* resistance used the CRISPR/Cas9 gene editing system to mutate the susceptibility gene *OsHPP04* in rice, resulting in improved resistance to the nematode, without affecting desirable agronomic traits [97]. Consequently, the immune responses in the “transgene-free” homozygous mutants of rice plants triggered by flg22, involving ROS burst, defense-related gene expression, and callose deposition, were raised. Additionally, not only were two “transgene-free” mutant lines (TF-H4-1 and TF-H6-1) significantly (*p* < 0.05) more resistant to *M. graminicola*, as the average number of adult females and total nematodes was reduced by 37.4–42.6% and 39.6–41.9%, respectively, but also all homozygous mutant lines with transgenic elements (H1-1, H2-1, H3-1, H5-1 and H5-2) exhibited a higher resistance to *M. graminicola* compared to the wild-type Nipponbare rice [97]. Such responses showed potential in developing PPN-resistant crops to contribute to durable agriculture and crop protection. Moreover, CRISPR-Cas9-mediated knockout of the *GhiMLO3* gene in cotton reduced the reproduction of the reniform nematode *Rotylenchulus reniformis* [98]. These genetic modifications in crops create “transgene-free” resistant plants that do not suffer from reduced growth or yield, providing a sustainable alternative to chemical nematicides [99].

Finally, the above-mentioned studies provided valuable insights into the systemic response of plants to PPNs and microbiomes. They offer better evidence summarization of current trends to activate/fortify plant immune systems by PPN-resistant genotypes and microbiomes. Yet, more exploration on exact recognition and signaling pathways linked to compounds/genes required for *R* operations and plant immunity is still needed. That is because factors impacting plant yield, microbiome, and PPNs are complex and interdependent. Other plant pathogens, insect herbivores, soil-borne microbes, and soil nutrition may influence yield directly and by affecting each other through indirect pathways. Further grasping of related microbiome scenarios is desperately needed to optimize their role.

### 3.4. Exploiting and Optimizing Microbiome Role Against PPN Species

Ambitiously conceived paradigm research efforts should exploit sophisticated tools empowered by unbounded enthusiasm for biotechnology to unravel the relevant molecular mechanisms. Beneficial roles of microbiomes against PPNs are not yet widely practiced in conventional agriculture, but recent results help to develop their optimum effectiveness. Advancing their efficacy will eventually lead toward their expanded field applications against not only PPNs but probably other soil-borne pests as well. Exploiting the extraordinary potentials that microbiomes offer as environmentally safe, effective, and durable alternatives to synthetic nematicides is quite possible (Figure 3). Thus, this is a timely opportunity for a review of our current knowledge, existing gaps, and the challenges ahead in order to upgrade the microbiome’s role against PPN species. Beneficial microbes possess a wide array of strategies to target both motile and sedentary PPNs’ life stages [106] to be fully exploited. However, such strategies are so diverse; their details are beyond the scope of the current review. Therefore, the focus herein is to exploit and improve emerging strategies related to the microbiome’s ability to prime and boost plant defense against PPNs. Yet, as these microbiomes can finally act as plant health promoters and BCAs against serious PPNs, their role could be astutely expanded for large-scale application.

#### 3.4.1. Grasp of Basic Factors Governing Composition and Activity of Microbiomes

The composition and activity of microbiomes are shaped by dynamic and multifarious interactions of ecological factors, host-derived constituents, and microbial interplay [107,108,109]. Assessments of such elements are good indicators of microbiome impact and diversity [109]. Likewise, biotic factors governing the composition and activity of microbiomes should be absorbed to manipulate their improved contribution to plant defense/health. Thus, the aforementioned interconnection between mechanistic and holistic approaches is materialized herein again. Root bacterial profile was examined using sequencing of the bacterial 16S gene across different *H. glycines*-susceptible and resistant soybean genotypes. The change in the microbial community under *H. glycines* infection indicated that this profile of susceptible and resistant soybean roots and their soil samples encompassed different bacterial communities, though slightly overlapped [107]. This study confirmed that the host genotype had a significant effect on the diversity of the root microbiome under *H. glycines* pressure in the greenhouse. The authors speculated that such differences may potentially explain the impact of beneficial bacteria and/or secondary effects relevant to *H. glycines* resistance. Hanif et al. [109] gave examples to confirm that the microbiome composition varies notably due to varied plant species and/or native soils. Clearly, diverse microbial populations may exist even in closely located plants of phylogenetically distant species. Moreover, distinct microbial abundances have been linked to specific plant genotypes, e.g., chromosomal substitutions in wheat varieties could affect rhizosphere microflora [110]. Furthermore, the stage of plant development [111] and genetic modifications like introducing specific genes [112] could change microbial communities, as shown in *Arabidopsis thaliana* (L.) Heynh. (Brassicales: Brassicaceae). On the other hand, plant exudates like organic acids, phenolic compounds, amino acids, and sugars as well as volatile compounds significantly impact microbial colonization and diversity [109]. In contrast, microbial metabolites contain compounds that may enhance the benefits of applying biocontrol strategies [14,113,114]. To sum up, shifts in nematode population densities occur directly/indirectly not only by the impact of abiotic factors but also by their hosts and enemies, biotic factors [1,75,115]. So, grasping terms like NAMPs, PRRs, and RLKs in the context of these related basics for plant immunity can help to leverage microbiome community composition and activity. These fundamentals should act as backgrounds for the transformational technologies needed to adopt an upgraded role for the microbiome in PPN control.

#### 3.4.2. Manipulation of the Plant Microbiome to Prime and Immunize Plants Against PPNs

*A*. *Leveraging Biotic and Abiotic Factors in Related Settings*

With the identification of fundamental factors that rule composition and activity of microbiomes, careful screening of beneficial microbial strains under controlled conditions should comply with such basics before microbial inoculation. The magnitude of the implied task is huge and sometimes might not be realistic. Because biotic/abiotic factors are mostly more sensitive in biocontrol tactics than chemical pesticides, it becomes increasingly important to quantify relevant variables. Pros and cons of common quantitative models used to characterize related patterns of these variables were recently reviewed [115]. Usually, microbial strains/species that do well in lab assays undergo greenhouse tests followed by field estimations. Contrary to lab and greenhouse tests, such field evaluations may show variable results with inconsistent microbial efficacy, indicating a dire need to optimize settings (biotic/abiotic factors). That is because it is quite possible that competition of inoculants with native fauna or/and flora, suboptimal strain selection, and ecological variables can degrade inoculant performance against PPNs [116,117]. Therefore, microbial formulations of inoculants should consider both niche compatibility and cell viability for sustained colonization and perfect microbial function [108,116,118]. For instance, *B. amyloliquefaciens* strain FZB42 should be set early in an adapted niche in order to trigger effective plant defenses [118].

Emerging techniques like encapsulation, surfactant addition, seed coating, gel, and nanotechnology can enhance adherence to root surfaces, reduce biotic/abiotic stresses, and/or be inexpensive [109,119,120,121]. Using a 5% solution of Arabic gum could improve both adhesive capacity and distribution of *Bacillus* species on the outer parts of the treated strawberry roots just before transplanting [120]. Moreover, Ajijah et al. [122] stated that optimizing inoculant functions via proper biofilms on plant roots can generally help in thriving plants. They advocated that these biofilms can boost plant protection from not only PPNs but also other pathogens by improved root-colonization and competition with pathogenic microorganisms, stimulating SAR/ISR and producing antimicrobials effectively. Consequent boosted activities may also include inducing signal substrate production, managing phyto-hormone levels, enhancing protein generation, and regulating gene expression [106,122]. Eventually, more research is needed to evaluate the reliability of these techniques in defending plants against PPNs in various agricultural scenarios under actual field conditions. That is presumably because SAR is not an inherent microbial feature by itself but rather relies on the microbe’s interplay with the planted genotype, targeted PPN species/race, and other biotic/abiotic settings.

*B*. *Examining the Exact Interactions and Outputs of the Components*

Several advantages of using microbial consortia rather than single-strain inoculants can generally be offered to boost plant growth and defense against PPNs under various field settings [1]. These are as follows: (1) multiple activities of the consortia along the course of the plant-growing season; (2) ability to function against more than one stage in the nematode life cycle; (3) diverse mechanisms to control PPN species/races; (4) various ecological niches of the used consortia that may boost consistency in biocontrol activities over a wide profile of soil conditions; and (5) potential to encompass different microbial species/races that negatively affect more than one PPN species/race simultaneously, thus improving the spectrum of the used inoculants [123]. The latter authors gave numerous related practical examples. To this end, improving the multi-functionality and stability of combined inoculants is better harnessed via integrating metabolic complementarity with other useful traits. For example, this integration can allow for functional variation, composition, and redundancy in addition to having systematical inoculation impacts on beneficiary soils and/or plants [75,124]. Abd-Elgawad [1] stressed that microbial consumption of various resources in dynamic metabolic strategies is more likely to achieve not only merged vigor but also harmonized combinations. This is especially important as time-related variation in activities among microbial communities in/on the plant roots/rhizosphere can lead to synergistic or additive effects.Thus, soil amendments combined with different *Trichoderma* spp. caused higher reduction in *M. incognita* population levels than those obtained by single ones [13]. Several studies [107,108,109,110,111,112] appreciated tailoring of consortia to specific ecological conditions and plant genotypes/developmental stage to improve their efficacy. Ultimately, the beneficial and well-synthesized microbiome should be harnessed to reliably work in multiple useful directions and for a longer period of time.

On the contrary, antagonism between BCAs in the microbial consortia or products through competition and antibiosis may reduce their activities. Single treatments as seedling drenches by *Burkholderia cepacia* strains Bc-2 and Bc-F and of *Trichoderma virens* strain Gl-3 significantly reduced numbers of *M. incognita* eggs + J2 per gm bell pepper root. However, these nematode parameters—eggs + J2—were not significantly affected from combining these treatments [125]. Furthermore, a single BCA species/strain may be more effective against PPNs than two other merged BCAs. A single fungal strain (*P. lilacinum* MR20) could outperform a mixture of two plant-growth-promoting bacterial strains (*Pseudomonas fluorescens* MR12 and *Serratia marcescens* MR25) in minimizing *M. incognita* population levels on tomato plants [126]. Moreover, efficacy of an individual species/strain in a microbial consortium may greatly vary, even in some commercial products that are documented to act as a good plant-defense biostimulant against PPNs. For instance, a single component (*B. subtilis*) in a commercial product referred to as Myco increased *M. incognita* infection indicators at any tested bacterial density; however, the whole microbial consortium of Myco proved to activate plant immune response and prime tomato plants against RKNs [2]. The authors advocated that differences in microbial performance may depend on the used species/strains as well as methods and doses of the application. Therefore, Myco proved to be reliable for inducing resistance against RKNs but only in a dose-dependent manner [127].

In the opposite direction, specific microbiota naturally linked to PPNs may protect them against microbial antagonists [128]. Recently, Trejo-Meléndez and Contreras-Garduño [129] underscored the essential functions of some PPN-linked microbial communities in defeating ecological hurdles facing the nematodes. Their roles may include aiding in exploiting the plant by suppressing or evading plant defenses [129]. Such mixed results in the role of microbes should be considered in manipulating PPN-infested soil. Microbial inoculants should be well-tailored to improve the synthesized microbiome for priming and defending plants against PPNs. Products of such live microbes are handled carefully, as they are more sensitive to biotic/abiotic stresses than those with only bioactive compounds. Such non-living products require less strict handling; e.g., they do not need refrigeration.

Ultimately, the exact interactions among inoculant components or with biotic/abiotic factors in intimate contact with the inocula are preferably done on a case-by-case basis to avoid negative or erratic outputs. For instance, the fitness impacts of numerous AMF symbionts remain context-dependent and can change due to different biotic/abiotic factors, e.g., new host backgrounds or existing fauna/flora that intricate predictions of their long-term dynamics. Otherwise, an approach that explicitly incorporates context-dependent factors in SAR array is direly needed. It enhances the predictability of SAR induction under definite field conditions.

*C*. *Harnessing Plant Microbiomes to Prime and Immunize Plants via Current Strategies*

Common practices such as cover cropping, crop rotation, soil mulching, applying biologicals/their bioactive compounds, and organic amendments should be upgraded in parallel to recent advances in grasping the tri-trophic interactions. The upgrading would set the interactions (embracing host plants, PPN species, and natural enemies) to boost proliferation of useful microbial taxa and/or suppressing PPNs within comprehensive strategies [1,75]. Thus, techniques to exploit microbially induced resistance would be carried out via exogenously added inputs to the required crops and (or) steering the native rhizosphere microbiome. Wang and Li [130] suggested certain crop rotations to build up positive soil legacies. These latter can steer soil microbiomes towards ones that benefit crop growth, e.g., by harboring a large fraction of biota able to induce ISR/SAR. Such soil legacies could be set via not only rotated crops but also cover crops [131]. In addition, incorporating specific soil amendments can serve plant microbiomes in sophisticated strategies. For example, added cow manure [containing 0.65% total nitrogen, 21.2 carbon to nitrogen (C/N) ratio] and poultry manure (0.72% total nitrogen, 20.1 C/N ratio) to two Egyptian farms of El-Ismailia governorate resulted in almost zero counts of RKNs in loamy sand soils, whereas RKNs could thrive in ten other farms. Higher levels of recorded organic matter and microbial counts, e.g., recorded *Trichoderma* spp., in the two farms than in the other ones were likely responsible for RKN suppression [132]. Differently, using species within a definite group of BCAs to simultaneously control pests/pathogens related to different taxa could be involved—for example, EPNs against both insect and nematode pests [15,133]. In such cases, advanced PCR-based techniques could differentiate closely related EPN species to distinguish their specific phenotypes; e.g., host specificity, persistence, and habitat adaptation [134]. Although cell-free supernatant generated by the EPN-symbiotic bacterial culture of few species showed significant effects against RKNs [14], the dynamics of such interactions remain largely elusive for many other species. So, the merits of such techniques can accurately uncover how molecular processes are involved in such nematode control and plant strengthening under various settings. The expected results would boost the chances of superior PPN control. Following the same line of thinking, Han et al. [135] proposed effective, eco-friendly crop protection strategies. The strategies implement multidimensional management of PPNs and other pests. They adjusted spatial dimensions for the interactive impacts of soil–crop–pest–natural enemy networks on pest control. Meanwhile, other related dimensions deal with time, i.e., pest interactions over the whole crop season. As microbiome-related enhancing plant immunity is engaged, adopting green IPM tactics like organic farming is fortified. They [136] aimed to enhance microbial diversity and abundance to improve ecosystem resilience and plant health, gaining ecological benefits too.

*D*. *Critical Synthesis to Operationalize the Optimization of Plant Microbiomes Against PPNs*

Interestingly, integrating innovations in common practices can develop superior safe and reliable PPN control, including relationships between plant microbiome and immunity. For example, useful top associations like optimizing root exudates or microbial metabolites, enhanced symbiosis, and plant-high responses to this symbiosis via classical plant breeding and/or targeted genome editing could be employed. Useful microbiomes can be successfully employed to enhance favorable performance of the plant defense [109,132]. While classical plant breeding and grafting to support plant defense against nematodes has recently succeeded in developing resistance in tobacco against *M. incognita* [29], Ibrahim et al. [46] suggested cloning and overexpressing the genes in charge of the PPN control task from the target and corresponding agents. They [46] used *Paecilomyces javanicus* (Friedrichs & Bally) A.H.S. Br. & G. Sm. (Eurotiales: Thermoascaceae) as an example to benefit plant protection efforts via fortifying its immune response against PPN infection. Many scientists are calling for GMO for climate-resilient plant genotypes [136]. However, a significant segment of consumers maintain a cautious or negative sentiment toward GMOs across food, feed, and industrial applications. They are largely driven by perceived health/environmental risks and a lack of trust in information sources. Using the commercial software’s sentiment analysis, 54% of the mentions were categorized as having a neutral sentiment, 32% as having a negative sentiment, and 14% as having a positive sentiment [137].

Importantly, intensive focus should be directed to tackle the fundamental differences in the results of relevant research, or at least to scientifically explaining their causes. A few examples of molecular techniques/devices are presented to vividly express their counterpart ones reckoning with updating. Treatments with *B. subtilis* increased *M. incognita* infection indicators at any tested bacterial cell density [2]. On the contrary, various beneficial roles of *B. subtilis* against PPNs, like inducing plant resistance, were recently documented [79]. This is probably because bacterial strains within the same species can have vastly different, and sometimes opposite, effects on soil nematodes. These effects may range from providing essential nutrients to causing death due to significant micro-variation in their genomes, gene expression, and metabolic profiles [138]. To overcome this conflict and the like, an unambiguous, high-resolution, and definitive identification of bacterial strains within a species like Whole Genome Sequencing (WGS) is considered the current gold standard [139]. Likewise, for related fungi, different AMF isolates may have different colonization abilities/efficiency. Moreover, the colonization of microbiome-generating formulations may be mediated by the diverse and abundant microbial communities associated with mycorrhizal roots, spores, and extra-radical hyphae. Variation in such attributes for different fungal strains/isolates of a single fungal species can affect the consistency of the tested molecular mechanisms especially when linked to field relevance. Fine-scale taxonomic resolution such as WGS, Multilocus Sequence Typing (MLST), or Microarray/Strain-specific PCR is typically required [140].

With remaining controversy, ISR is thought to be acquired upon local induction by beneficial microbes, while SAR is assumingly acquired upon local induction by a pathogen. Recent research has often proven that the boundaries between these two pathways are mostly fluid [2,141]. Microbiomes (non-pathogens) can induce SAR via several ways [142]. These involve pathway activation (definite bacteria proved to trigger SA-dependent pathway and express PR proteins), strain-specific responses (e.g., *Bacillus cereus* AR156 can simultaneously activate both SAR (SA-dependent) and ISR (JA/ET-dependent) pathways), molecular mimicry (some microbes can produce MAMPs that are recognized by plant receptors in a manner similar to pathogen signals, leading to a full SAR-like systemic response), and finally some non-pathogenic rhizobacteria releasing volatile signals (e.g., 2,3-butanediol) that can induce a systemic resistance state throughout the plant, sometimes activating SAR-related genes in distal tissues. So, increased recognition that “SAR-like” responses induced by non-pathogenic microbes can be exploited to leverage microbiomes should be spread to enlighten growers for sustainable agriculture.

However, dissemination of these new molecular facts should be supported by relevant field practices to maximize the share of microbiomes in PPN management. These microbiomes are usually considered only as growth promoters or biostimulants because of the efficacy gape between laboratory and field results due to the relevant settings. To expand their uses, novel approaches may have dual purposes: locating the best field sites for effective PPN control by microbiome and convincing growers/stakeholders of the concrete microbiome-biocontrol value. Practically, statistical models of nematode-spatial patterns were used to optimize PPN sampling procedures and assess certain field sites for controlling *Meloidogyne hapla* and *Pratylenchus penetrans* in commercial potato production [141]. Consequently, hotspot infections by the PPN species were determined. This technique may similarly be used to save the costs of PPN control if the microbiome is strategically applied only for such spot treatment. It can also enable the best seed-location matching, using proper rates of the applied bionematicides, and reveal relationships between beneficial/harmful organisms in space and time for superior IPM schemes. To circumvent limitations related to these models, Abd-Elgawad [116] discussed merging sophisticated techniques such as PCR-based approaches to identify and quantify species, bioinformatics for comprehensive modeling, and volatile organic compounds as signals for soil inhabitants.

Yet, to offer a paradigm shift and expedite detection/count processes with objectivity/accuracy, automated nematode/related microbes have been developed [143]. In another system, a smart soil organism detector can rapidly isolate, extract, count, identify, and separate soil organisms in a high-throughput, accurate, and reproducible mode [144]. It is well known that the more PPN/related microbial samples collected, or bioassays run, the longer it takes to count. So, in addition to saving time and effort, these are key tools for better understanding global soil biodiversity.

*E*. *Widening the Microbiome Role Against More Key PPNs and over Two Generations*

**Figure 4:**
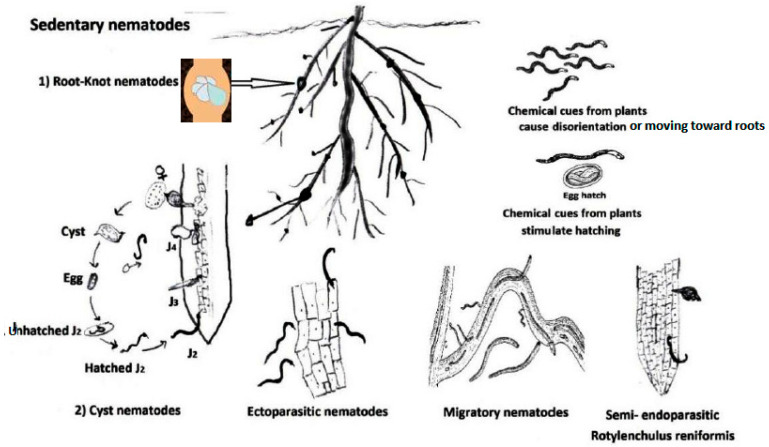
Categories of soil plant-parasitic nematodes showing different harmful interactions with their host plants. Beneficial microorganisms should be harnessed to combat such categories.

Current research on related microbiome activities is mostly focused against endoparasitic sedentary nematode species. It is preferable to stretch the microbiome role against other key groups of PPN species (Figure 4). For instance, SA biosynthesis genes needed to induce SAR in plants against PPNs were recorded to be upregulated by the fungus *P. lilacinum* [145]. Thus, this fungus at 10^8^ conidia ml^−1^ could consistently suppress populations of the rice white tip nematode, *Aphelenchoides besseyi* Christie (Tylenchida: Aphelenchoididae) [146]. Further and related research against this and other PPN species is needed. Vicente et al. [147] speculated that a specific group of Ophiostomatales fungi (genera *Leptographium*, *Ophiostoma*, and *Graphilbum*) possess such an adaptive reaction with the pinewood nematode *Bursaphelenchus xylophilus* Steiner & Buhrer (Nickle) (Aphelenchida: Parasitaphelenchidae) that they can significantly diminish the nematode population levels. *Leptographium* isolates showed more promising results for PWN control than the others. They outcompeted the other species, especially *O. ips*, and significantly suppressed development of *B. xylophilus* populations, suggesting this to be a natural antagonist not only for the other blue-stain species but also for the nematode. Their results for the functional role of fungal communities inhabiting *B. xylophilus*-infected trees can lead to identifying key points in the nematode life cycle to disrupt the disease cycle via more natural alternatives. Exploring the molecular adaptation of such specific fungi and other bacterial endophytes [148] associated with *Pinus* species to optimize their biocontrol potential against *B. xylophilus* is suggested. Of special interest, Kim et al. [149] examined the potential of chosen pine endophytic bacteria to induce resistance against *B. xylophilus*. Their study could identify bacterial strains that triggered defense responses in the infected pine trees through their ability to induce the expression of the *PR10* gene, a marker of plant defense. Out of 92 bacterial strains, only 15 were identified to significantly enhance *PR10* expression in pine callus cultures. In addition, the interaction of *Ditylenchus dipsaci* Kuhn (Tylenchida: Anguinidae) and *F. oxysporum* on garlic led to less severe disease in the infected bulb and lower *D. dipsaci* population densities than when the plants were inoculated with the nematode alone [150]. The authors contemplated that suppression of *D. dipsaci* populations by the fungus may be attributed to the formations of physical barriers like a mycelial mat coating the basal plate, defense responses (e.g., cell wall thickening), or competition for available nutrients.

Colonization by *B. firmus* I-1582 on roots of *Arabidopsis thaliana* significantly suppressed *H. schachtii* reproduction, pathogenicity, and development over two generations [151]. While I-1582 was attracted by *A. thaliana* root exudates and colonized the roots via a strictly pH-dependent development, it protected the plant from infestation by *Heterodera schachtii*. To get an indication of the bacterial mechanism, infection assays were performed in the presence of living bacterial cells (LBC), dead bacterial cells (DBC), or cell-free supernatant (CFS). The presence of living cells caused a significant decrease in the number of developed males, females, and total nematodes at 14 DPI, with average decreases of 17.7%, 26%, and 20.6% compared with the control, respectively. Inoculation with DBC only reduced male number by 19.1%, while CFS did not have any suppressive impact on *H. schachtii* parasitism. At 28 DPI, the females surrounded by LBC were significantly smaller in size (18.6%) compared with those of the control, while the females developed at the bacterium-treated plants that were not colonized by LBC were not different in size compared with the females at the control plants. Moreover, DBC and CFS treatment did not influence female development. None of the treatments had an impact on the female-associated feeding site. Moreover, the cysts that developed at the end of the second-generation infection were analyzed for presence of bacteria. The attachment of bacteria in the “without LBC” group of the first-generation cysts was not visible under the microscope, but the amount of bacteria “with LBC” attached to the second-generation cysts was quantifiable. On average, 8 and 5 CFUs could be determined on the second-generation cysts that developed from juveniles that hatched from the first-generation cysts of the “LBC” group and the “without LBC” group, respectively. Thus, it was concluded that the two groups represent first-generation cysts with a different bacterial load and those with “LBC” can persist for longer time [151]. Ahmed et al. [152] proved that the *Bacillus cereus* Frankland & Frankland (Bacillales: Bacillaceae) strain B48 significantly reduced *Heterodera avenae* Wollenweber (Tylenchida: Heteroderidae)-white female development with enhanced wheat root length of infected plants relative to the untreated check. The authors confirmed the possible strain’s ability to induce systemic resistance in the plant system for protection from various soil-borne PPNs. Another possibility is that this rhizospheric *Bacillus* strain exhibited hyperplastic activity and by excreting hydrolytic enzymes can easily attack the nematodes and penetrate their cuticle.

In another study, the effect of AMF *Glomus etunicatum* (Glomerales: Glomeraceae) W.N. Becker & Gerd. on *H. glycines* resulted in a 28% reduction in nematode females in the soybean root system of mycorrhizal plants compared to untreated roots, suggesting that AMF protected the host plant against *H. glycines* [153]. They suggested that AMF *G. etunicatum* acts indirectly, promoting the nutritional plant status that creates tolerance to the presence of the pathogen by soybean. Besides mechanisms such as improved plant nutrition and competition, experimental evidence supports a major role of mycorrhiza-induced resistance and priming of plant defenses in the observed protection [154]. During the mycorrhiza set, modulation of the aforementioned plant defense responses occurred, thus attaining a functional symbiosis. As a consequence of this modulation, a mild but effective activation of the plant immune responses seems to occur, not only locally but also systemically [154]. Thus, defense responses are coordinated by small molecules that act as signal transducers and tailor the coordinated expression of genes that code for defense-related proteins and compounds. Among these molecules, JA, SA, and ET play key roles. Yet, according to the pathogen lifestyle, one signaling pathway will prevail over the others. For PPNs, it is generally assumed that the SA-dependent pathway regulates responses such as programmed cell death and is effective against biotrophic organisms like PPNs [52,154]. As *Xiphinema index* Thorne & Allen (Dorylaimida: Longidoridae) is a serious pest that causes severe damage to the root system of grapevines and is a vector of grapevine fanleaf virus, AMF (*Glomus intraradices* BEG141) was used for its control [155]. The mycorrhiza formation reduced both the *X. index* number in the soil and its gall formation on the roots of the grapevine. Such suppressive effects increased with time and were greater when the nematode was post-inoculated rather than co-inoculated with *G. intraradices*. Using a split-root system, decreased *X. index* development was shown in mycorrhizal and non-mycorrhizal parts of mycorrhizal root systems, indicating that both local and systemic induced bioprotection mechanisms were active against the ectoparasitic nematode. Expression analyses of expressed sequence tags generated in a subtractive suppressive hybridization library, representing plant genes upregulated during mycorrhiza-induced control of *X. index*, and of described grapevine defense genes showed activation of chitinase 1b, pathogenesis-related 10, glutathione S-transferase, stilbene synthase 1, 5-enolpyruvyl shikimate-3-phosphate synthase, and a heat shock protein 70-interacting protein in association with the noted local and/or systemic induced bioprotection against *X. index*. These data suggest priming of grapevine defense responses by the *G. intraradices* and transmission of a plant-mediated signal to non-mycorrhizal tissues. Grapevine gene responses during the AM-induced local and systemic bioprotection against *X. index* point to biological processes that are related either to direct effects on the nematode or to protection against *X. index*-imposed stress to maintain root tissue integrity [155].

Moreover, AMF significantly reduced *M. incognita* penetration through altered root exudation of their tomato host [156]. The mycorrhizal (*Glomus mosseae*) effect temporarily paralyzed nematodes too, compared with application of water or non-mycorrhizal root exudates. Yet, *M. incognita* juveniles resumed motility in water at 2 days after root exudates application, possibly due to the degradation of active compounds in the exudates or the lack of inflow of new root exudates. This explanation was supported by the motility-inhibiting effect that was prolonged by transferring the nematodes to a fresh solution of mycorrhizal root exudates.

When testing biological induction of resistance to *Globodera tabacum solanacearum* Lownsbery & Lownsbery (Tylenchida: Heteroderidae) in flue-cured tobacco [*Nicotiana tabacum* L. (Solanales: Solanaceae)], Parkunan et al. [157] found that two *Bacillus* strains—among four rhizobacterial combinations—had the most consistent effect in decreasing the nematode cysts under greenhouse and field conditions. Other *Bacillus* species showed similar adverse effects on *Rotylenchulus reniformis* Linford & Oliveira (Tylenchida: Hoplolaimidae) infecting soybean plants [158]. Additional species like *B. thuringiensis* were reported to protect plants from *X. index* infecting grapevine [159]. For other key PPN and bacterial genera, the endophytic bacterium *Serratia ureilytica* Bhadra et al. (Enterobacterales: Yersiniaceae) could negatively affect *Nacobbus aberrans* Thorne (Tylenchida: Pratylenchidae) on Chili plants. This bacterium significantly reduced the number of galls, egg masses, eggs, and reproduction factor of the nematode on the plant roots, compared to the control where *S. ureilytica* was not applied [160]. Mycorrhiza-induced resistance has been shown to be efficient against other important nematode species such as *Radopholus similis* and *Pratylenchus coffeae* (Zimmermann) Filipjev & Schuurmans Stekhoven (Tylenchida: Pratylenchidae) infecting banana [161]. Therefore, AMF are being developed for commercial applications, e.g., Ectovit^®^, Symbivit^®^, and Rhodvit^®^ [162].

Notwithstanding the utility of microbiomes against such key PPN species, the molecular—genetic/epigenetic—mechanisms by which the related plant immunity is triggered need further research to leverage and expand their usage. Priority research areas can also merge plant microbiome profiling with breeding techniques to assemble superior microbiomes against PPNs. Other areas are to optimize advantageous effects of root exudates on the microbiomes. Moreover, research is needed to accurately examine the impact of specific inoculants or treatments such as immune hormones, especially SA, JA, and ET, on the composition of microbiomes and how microbiomes shift the plant immune system. Eventually, understanding the related ecology, biology, mode of action, and interactions of these microbiomes with other agricultural inputs is still needed for future application against key PPN species in sustainable agriculture.

## 4. Microbiome Merits Serve to Raise Awareness and Increase Its Adoption

Currently, the consensus is that there is no single PPN control method that has all the relevant merits, that is, effective, inclusive, economical, and safe for non-targets [163]. A beneficial microbiome that raises plant immunity and offers defense priming is not an exception regarding this fact, i.e., the microbiome occasionally provides only partial plant protection. Thus, its integration with other PPN control method(s) is preferable. In contrast, the microbiome can contribute to immunizing plants for the long term, and plant priming may persist even after removal of the priming agent [164]. That is because plants have a non-adaptive immune system that is set by molecular shifts. Epigenetic modification implied by induced resistance priming can enable plants to acquire a memory of stressful conditions. Consequently, it leads to changes in gene expression. Such changes are heritable to the plant progeny, resulting in maintenance of priming up to the next generation [165]. Analysis of gene expression suggested that tomato plants pre-exposed to Gram-positive bacteria isolated from root nodules exhibited stronger jasmonate-mediated defense responses compared to non-primed plants upon the attack of a pathogen, which resulted in long-term induced systemic resistance to pathogen attack [166]. Moreover, the symbiotic association of AM fungi and date palm resulted in reduced oxidative damage during long-term drought stress via enhanced activity of antioxidant enzymes CAT, SOD, APX, and G-POD. Thus, AM symbiosis reduced the detrimental effect of long-term drought stress on the growth parameters of seedlings [167]. Several studies proved that defense priming can be inherited; this phenomenon is referred to as trans-generational immune priming or trans-generational memories of plant defense. Progeny of the parental plants primed by treatment with beta-aminobutyric acid or infection with avirulent *Pseudomonas syringae* bacteria indicated increased expression of SA-dependent defense genes and stronger resistance to infection by both virulent *P. syringae* and the downy mildew pathogen *Hyaloperonospora arabidopsidis* [168,169]. This has set priming of the progeny generation when parental *Arabidopsis* plants have been subjected to several challenges with virulent bacteria, *P. syringae*. In these studies, not only was the primed state passed to the immediate offspring generation, but increased disease resistance could also be detected in the grandchildren of the original infected plants and was therefore inherited over one stress-free generation. In another study, the next generation of tomato plants primed with *Trichoderma atroviride* displayed improved resistance to RKN, *M. javanica*, without any cost in terms of enhanced plant size [170]. A direct activity of *T. atroviride* against *M. javanica*, in terms of 42% reduction in the number of galls, 60% in the number of egg masses, and 90% in the number of adult nematodes inside the roots, was noted in tomato grown under greenhouse conditions [170]. Further, an in vivo split-root system demonstrated that *T. atroviride* induces systemic resistance towards *M. javanica*, without the need for the organisms to be in direct contact, and significantly reduced the number of galls (20%) and adult nematodes inside tomato roots (87%). The first generation (F1) of *T. atroviride*-primed tomato plants inherited resistance to RKN, although the induction of defenses occurred through different mechanisms, and in varying degrees, depending on the *T. atroviride*–*M. javanica* interaction. Plant growth promotion induced by *T. atroviride* was inherited without compromising the level of resistance to *M. javanica*, as the progeny of *T. atroviride*-primed plants displayed increased size and resistance to *M. javanica* without fitness costs. Thus, F1 tomato plants from the five split-root tests showed phenotypic differences among them. When the green mass of F1 plants was calculated and the value of 100% was assigned to the green mass coming from the F1 plants (control), F1 plants derived from *T. atroviride* and *T. atroviride* + *M. javanica* treatments were 130 and 125% relative to the control, respectively. Gene expression results from the defense inductions in the offspring of *T. atroviride*-primed plants suggested that auxin-induced ROS production boosted by *T. atroviride* may act as a major defense strategy during plant growth [170]. Tiwari et al. [164] assured that such a microbiome merit in plant immunological memory should be added to other related ones. These include its wide-spectrum impact, low fitness cost, and long-lasting durability, which make it attractive for sustainable crop protection. On the contrary, plant (*R*) genes encode immune receptor proteins to induce defense responses that are mostly linked to a reduction in growth and yield, known as fitness costs [171].

Commonly, nematode suppression in soil can offer a quick and non-specific defensive line for other pathogens too. Yet, plant specific suppression of PPNs needs time to react, and it has a memory related to a specific invading pathogen [52,129]. Thus, to get more benefits of the soil microbiome and/or upgrade plant defense, activation of the microbial community interfering with the specific PPN infection may be initiated or shortened by putting in the “microbial or material kick start.” This start could be via mulching the soil [6], adding supplementary food sources for the microbiome [172], or applying useful endophytes solely [173] or merged with a plant extract or other materials [1,26]. Moreover, pre-treating plant roots with favorable microbial communities or proper hormone generators could activate plant defense [51].

Limitations in expanding such processes/merits mainly stem from the low magnitude of these interventions and their inconsistent effects. In addition, the lack of awareness of efficient implementation methods by stakeholders, especially resource-poor farmers, is a contributing factor. Limited knowledge of these beneficial microbiomes by extension personnel who advise growers usually aggravates the matter. To optimize household food security and concurrently improve their crop yields, related sustainable, low-input, and environmentally friendly PPN management strategies have to be simply applied and adapted to suit their needs. This concept has been discussed in the frame of conserving and enhancing biocontrol of PPNs [173] and theoretically presented to improve the adoption of green IPM tactics [135]. Nonetheless, thus far, it has not been comprehensively attempted in earnest. This could be achieved by means of innovative and applied research by appropriately trained nematologists and biologists. They can train, assist, and guide extension officers and farmers to enhance the quality and quantity of their produce by minimizing the adverse effect of the PPNs in their crops. Therefore, relevant efforts for enlightening stakeholders will facilitate expanding the benefits of ISR/SAR-inducing microbes to extension agents and farmers. Moreover, improving commercial syntheses and formulations of related products and their application efficacy should be a continuous process. They should facilitate incorporating the beneficial microbiome into IPM plans. As complementary tools for PPN control, plant microbiome and immunity interactions can be exploited for very efficient uses that maintain the environment, do not pollute, and secure the quality/quantity of crops in an inexpensive manner. These activities will continually broaden the boundaries of sophisticated approaches and scalability of pathogen/pest management while boosting the crop health and yield.

## 5. Conclusions

It is well established that plants respond to PPN infection using a two-branched immune system. These branches are pattern-triggered immunity and effector-triggered immunity and are defined by the types of molecules recognized by plants as indicators of a PPN attack. The first branch interacts with molecules common to many microbes, involving even non-pathogens, and its immunity is defeated in PPN-susceptible plants. Their defense is suppressed via downregulation of the genes engaged in SA-mediated defense, very early in plant–PPN interaction. The second responds to pathogen virulence factors, either directly or via their effects on host targets. It acts using the polymorphic nucleotide binding-LRR protein products encoded by most *R* genes. Techniques like classical breeding, grafting, genetic engineering, and genome editing are ongoing to create genotypes with resistance to PPNs. Yet, a few related drawbacks of *R* genes need to be overcome. This would be done via exploring more resistance genes, their activation mechanisms, and signaling networks in the context of offering a more holistic recognition of plant defense for managing key pathogens/pests. Moreover, using multiple resistance genes as major *R*-genes and minor genes within IPM to decelerate the evolution of resistance-breaking pathotypes is necessary. Remarkably, microbiome-triggered immunity is coming to the forefront with numerous and diverse research projects. These microbiomes that can prime and immunize plants against pests like PPNs involve AMF, PGPR, and other biocontrol fungi. Their systemic immunization against PPNs is chiefly mediated by upregulation of SA-dependent defense genes, typically *PR*-genes. Beneficial relationships between plant microbiome and molecular immunity can contribute in providing safe and reliable PPN control strategies. Therefore, careful steering of the indigenous rhizosphere microbiomes and/or adding relevant inoculants should be followed to optimize PPN management. Within this context, it is critical to properly activate SAR and/or ISR via the related factors. These latter comprise the involved strains of the microbiome, plant-genetic constitution, manipulating the existing fauna/flora, and compatibility with the other engaged abiotic elements, as well as techniques and doses/concentrations of the inoculants. Further research is required on such factors on a case-by-case basis to avoid unpredictable results. Sustainable IPM plans would preferably involve immunizing plants against not only endoparasitic sedentary nematodes but also more key PPN species and/or other pests in a holistic approach. The end in view is to change the microbial processes occurring mostly at the microscales, molecular biology scales, into large-scale application for enhancing crop yields. Thus, improved formulations and applications of products containing such microbiomes should be earnestly attempted. Meanwhile, agricultural extension workers would simplify relevant information and the merits of their usage for their familiar adoption by stakeholders.

## Figures and Tables

**Table 1:** Examples of protein effectors divided by families within different fungal genera and species inducing plant-systemic resistance *.

Family	Protein	Fungal Species	Role in Interaction with Plants	Reference
Glycoside-hydrolases	Crh1	*Trichoderma harzianum*	Plant defense elicitors in *Trichoderma*–plant interactions	[86]
	FoEG1	*Fusarium oxysporum*	triggering cell death and inducing plant defense responses	[87]
	Thph1 and Thph2 (cellulase-like protein)	*T. harzianum*	Inducting defense-related genes	[88]
Cerato-platanins	Sm1	*T. harzianum*	Proteinaceous elicitor of plant host resistance	[86]
	Sm2 (small protein 2)	*T. virens*	Engaged in colonizing roots and protecting the plant	[89]
	Cpe1	*T. longibrachiatum*	Inducing plant disease resistance	[90]
Hydrophobins	Hyd1	*T. harzianum*	Inducing maize systemic resistance	[91]
	HYTLO1	*T. longibrachiatum*	Stimulate defense-related responses and boosting growth	[92]
	VdHP1	*Verticillium dahliae*	Inducing cell death and activate plant immune responses	[93]

* More details found in the related references for the fungal strains and their functions against the pathogenic species.

**Table 2:** Molecular transcription factors (TF) engaged in plant responses to invasion and development of five key plant-parasitic nematode species.

TF	Plant	Gene/Genotype/TF	Function/Nematode Species	Ref.
WRKY	*Arabidopsis thaliana*	AtWRKY23	Knocking down its expression lowered *Heterodera schachtii* infection	[100]
WOX11	*Arabidopsis thaliana*	LBD16 and other WOX genes	Forming lateral roots to reduce the impact of *H. schachtii* infections	[101]
GT-3a (a trihelix TF)	*Arabidopsis thaliana*	TOZ and RAD23C	Its stabilization by *Meloidogyne incognita* effectors led to suppressing related plant genes and easing the formation of the giant cells	[102]
SlWRKY3	*Solanum lycopersicum*	SlWRKY23	Upregulated resistance to *Meloidogyne javanica* via shikimate pathway activation	[103]
SLWRKY72a/SLWRKY72b	*S. lycopersicum*	Mi-1 induces effector-triggered immunity	Upregulating activator of immune response against *M. incognita*	[104]
WRKY	*Oryza sativa*	OsWRKY34, OsWRKY36, and OsWRKY62	Their overexpression suppresses the defense-related genes, implying that they function as a negative regulator of innate immunity against *Meloidogyne graminicola*	[103]
WRKY	*Oryza sativa*	OsWRKY62, OsWRKY70 and OsWRKY11	Their early upregulation induced oxidative stress against *Hirschmanniella oryzae*, that is, upregulated 3 Peroxidase precursors and 2 glutathione S-transferases	[105]

## Data Availability

The original contributions presented in this study are included in the article. Further inquiries can be directed to the corresponding author.
